# Accuracy of reduction and postoperative symmetry assessment of customized z-shaped miniplates versus conventional miniplates in mandibular fractures treatment: a randomized controlled clinical trial

**DOI:** 10.1186/s12903-026-08555-5

**Published:** 2026-05-18

**Authors:** Mariam A. Abd EL Hamid, Ahmed S. EL Mahallawy, Mohamed E. Saber

**Affiliations:** 1 Faculty of dentistry, Alexandria University, Champollion St., Azarita, Alexandria City, 21527 Egypt; 2 Oral and Maxillofacial Surgery Department, Faculty of Dentistry, Alexandria University, Alexandria City, Egypt

**Keywords:** Mandibular fractures, Computed tomography-based, Miniplates, Digital assessment, Symmetry, Operative time

## Abstract

**Background:**

Mandibular fractures are common facial injuries requiring precise reduction to restore function and aesthetics. Conventional fixation methods not always achieve optimal postoperative symmetry.

**Aim of the study:**

To objectively evaluate and compare the radiological outcomes of customized Z-shaped miniplates versus conventional two-miniplate fixation using five computed tomography -based digital methods. Also, operative time was recorded.

**Materials and methods:**

A prospective, parallel, randomized controlled superiority trial included 22 patients of both genders, with unilateral parasymphyseal/body mandibular fractures. Allocation (customized Z-miniplates, *n* = 11; two miniplates, *n* = 11) was done by independent statistician. Mann-Whitney U, Wilcoxon-signed rank and Chi-square tests were applied (*p* < 0.05).

**Results:**

Mean operative time was significantly reduced at study group (*p* < 0.001). Symmetry quantitative assessment showed smaller postoperative differences in the study group (*p* < 0.05).

Symmetry evaluation by Asymmetry Index equation revealed significant improvement at points Co and La (*p* < 0.05), while points Go and MF showed no significant changes (*p* > 0.05).

Accuracy of reduction quantitative assessment demonstrated significant smaller postoperative values in study group (*p* < 0.05).

Evaluation of reduction accuracy by chromatographical analysis (quantitatively) showed smaller values at all bilateral landmarks in study group (*p* < 0.05), except La point on fractured side (*p* = 0.3), while (qualitatively) showed more green areas in study group.

**Conclusion:**

Customized Z miniplates demonstrated superior postoperative symmetry, higher reduction accuracy and shorter operative time compared with conventional miniplates. Given the sample size, these findings should be interpreted as preliminary evidence supporting potential clinical efficiency of customized Z miniplates in the management of parasymphyseal/body mandibular fractures.

**Trial Registration:**

This study is retrospectively registered at clinicaltrials.gov with initial release at 06/27/2025 under the number (NCT07094867), Where the trial protocol and statistical analysis plan can be accessed.

## Introduction

One of the most common fractured bones in the face is the mandible, with incidence ranges from 36% to 59% of all facial fractures [1, 2]. The most common causes include road traffic accidents, sports injuries, interpersonal violence and falls from heights [3].

Mandibular fractures are classified based on location into symphysis, parasymphysis, body, angle, ramus, condyle, and coronoid processes [4].

Clinical presentation of mandibular fractures includes malocclusion, mandibular deformity affecting post-operative facial asymmetry, numbness of the lower lip due to damage to the mental nerve and dysphagia [2, 5].

The treatment of mandibular fractures has evolved over time starting with circumdental wires by Hippocrates [6] till the primary treatment include closed reduction or open reduction and internal fixation (ORIF) using plates and screws today [7].

Miniplate has become the standard method [8], However, because of higher complication rates and better understanding of mandibular biomechanics [7], three-dimensional (3D) plates have been developed by Farmand and Dupoirieux [9] providing more stability. However, Various studies in the literature suggest that 3D miniplate system is difficult to adapt and use in the fractures involving the mental nerve.

To overcome the shortcomings of the two-miniplate system and 3D plate, Prajwalit has proposed a design of Z shaped miniplate that may provide superior stability, support, easier in adaptation and preservation of mental nerve [10].

Recently, computer-aided design and manufacturing (CAD/CAM) technology has enabled the development of customized plates that adapt precisely to patient-specific anatomy to improve reduction accuracy, provide stable three-dimensional alignment and minimize intraoperative adjustments [10, 11].

By closely adapting to the native contour of the mandible, customized Z-shaped miniplates has been expected to enhance postoperative symmetry, reduce operative time, and potentially improve both functional and esthetic outcomes compared with conventional fixation methods.

Despite the increasing use of digital planning and customized fixation plates in mandibular fracture management, comparison between different geometries of miniplates regarding reduction accuracy and postoperative symmetry remains limited.

Therefore, this study aimed to compare postoperative symmetry, reduction accuracy, and operative time between customized Z-shaped miniplates and conventional two-miniplate fixation in parasymphyseal/body mandibular fractures.

The null hypothesis assumed that no significant differences would exist between the two fixation techniques.

## Materials and methods

Patients with parasymphyseal or body mandibular fractures indicated for surgery were recruited prospectively between April and December 2024 from cases admitted to the Emergency Department at Alexandria University Teaching Hospital. All patients were operated on and followed up at the Oral and Maxillofacial Surgery Department, Faculty of Dentistry, Alexandria University, Egypt.

Primary outcome was to compare the accuracy of reduction between customized Z miniplates and 2 miniplates, while secondary outcomes were post operative facial symmetry and operation time.

### Ethical compliance and registration

The study was conducted in accordance with the Helsinki Declaration (2013) and approved by the research Ethics Committee of Alexandria University Faculty of Dentistry, Egypt (IRB No. 001056 – IORG 0008839 with ethics no. 0866-02/2024) prior to any research-related activities. Informed consent was obtained from each patient prior to their enrollment and any surgical intervention after explaining the study objectives and procedures.

This study is retrospectively registered at clinicaltrials.gov (NCT07094867), with registration submitted on 27 June 2025. The first participant was enrolled on 20 April 2024 [1st operation on 22/4/2024 and last operation on 19/12/2024]. Retrospective registration occurred due to administrative and institutional procedures. The study protocol, eligibility criteria, randomization, primary and secondary outcomes measures were prespecified before first patient enrollment and were not changed throughout the trial in accordance with CONSORT guidelines. Nevertheless, retrospective registration represents a potential source of bias. No deviations from the registered protocol or outcome measures occurred during the conduct of the trial.

### Randomization and allocation

This study was a prospective, parallel, superiority randomized controlled clinical trial with a 1:1 (customized Z-miniplates, *n* = 11; two miniplates, *n* = 11) allocation ratio conducted in accordance with CONSORT guidelines. Participants were randomized to either study group (customized Z-shaped miniplate) or control group (conventional miniplate fixation) using block randomization (block size = 4). The allocation sequence was computer-generated (https://www.randomizer.org/) by an independent statistician and implemented using sequentially numbered, sealed opaque envelopes to ensure allocation concealment. After enrollment and baseline assessment, an independent coordinator opened the envelope to assign the intervention.

### Blinding and bias mitigation

Due to the nature of the surgical intervention, blinding of assessor was not feasible, as measurements were performed by a member of the surgical team. To minimize detection bias, CT datasets were anonymized and presented in randomized order without group labels. Measurement reliability was assessed, demonstrating excellent intra-examiner agreement (ICC ≥ 0.99) with narrow confidence intervals, statistical analyses were performed by a blinded statistician. While these measures strengthen the objectivity of the findings, the lack of full radiological blinding is acknowledged as a limitation.

Sample size estimation was performed assuming a 95% confidence level, 5% alpha error, and 80% study power. The mean immediate interfragmentary gap was reported as 0.89 ± 0.06 mm for the 3D customized plates and 1.08 ± 0.21 mm for the conventional mini plates, indicating accurate reduction as virtually planned [13]. Based on an independent means comparison and a pooled standard deviation of 0.14, a minimum of 10 participants per group was required to detect an effect size of 1.357, this number increased to 11 to make up for the 10% loss to follow up yielding a total of 22 participants (11 per group). Sample size was based on Rosner’s method calculated by G*Power 3.1.9.7.

(To our knowledge, there are currently no published studies that provide sample size estimation specifically for parasymphyseal/body mandibular fractures. Therefore, we used the closest available reference in the literature (Huang et al.) which provides relevant facial symmetry data to guide our calculation.).

### Eligibility criteria

The study included adults (20–42 years), of both genders, medically fit dentulous patients who accepted the digital surgery, with unilateral parasymphysis/body mandibular fractures (to ensure homogeneity of the fracture pattern and to avoid confounding factors that could influence outcomes) and disturbed occlusion. Fractures clinically and radiographically (CT confirmation) indicated for open reduction. The exclusion criterias included patients with comminuted, pathological, infected, non-displaced, mid facial and the other types of mandibular fractures.

### Materials

1. Customized titanium Z shaped miniplate: composed of 2 horizontal arms, connected by an oblique bar. Each arm with 4 screws holes with 2 mm diameter, inter-screw hole distance crossing fracture line is 8 mm while the distance between the 2 holes at each side of fracture line with is 4 mm. (Fig. 1)
  **Fig. 1:**
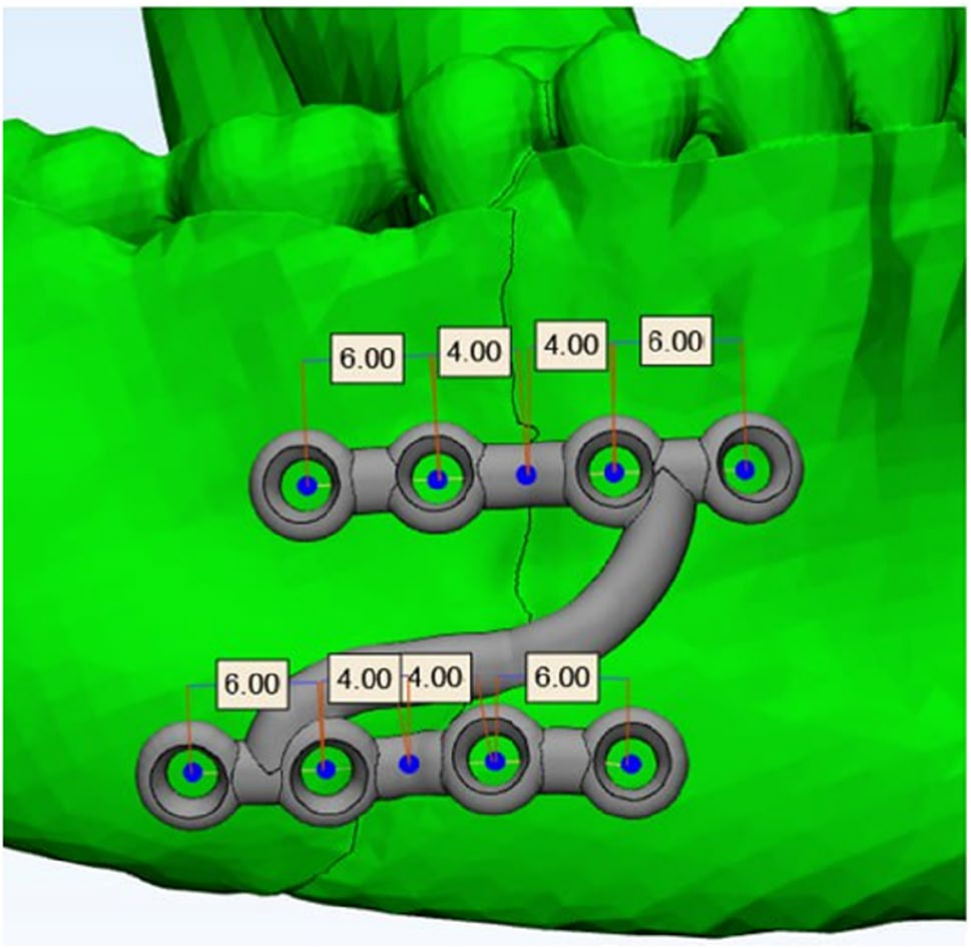
3D planning of the customized Z plate
2. Standard titanium miniplates.
3. Titanium screws 7- and 9-mm length & 2 mm head diameter.
4. Computed tomography DICOM file
5. (3-matic) Research 13.0 (x64) software.

#### Preoperative virtual planning

Virtual planning required approximately 4 h. Customized plate milling required approximately 24 h. The total interval between digital planning and surgery was standardized to approximately 48 h for all patients in both groups to ensure methodological consistency and avoided timing-related bias.

##### CT Acquisition

All CT scans were performed using the same Philips 16-detector CT scanner at baseline and at 6 months. CT parameters were standardized and matched for each patient’s follow up: tube voltage 100–130 kVp (depending on patient weight), tube current 150–175 mAs, slice thickness 1 mm, and images were reconstructed using a high-resolution bone kernel.

##### Virtual planning for study group

**Fig. 2:**
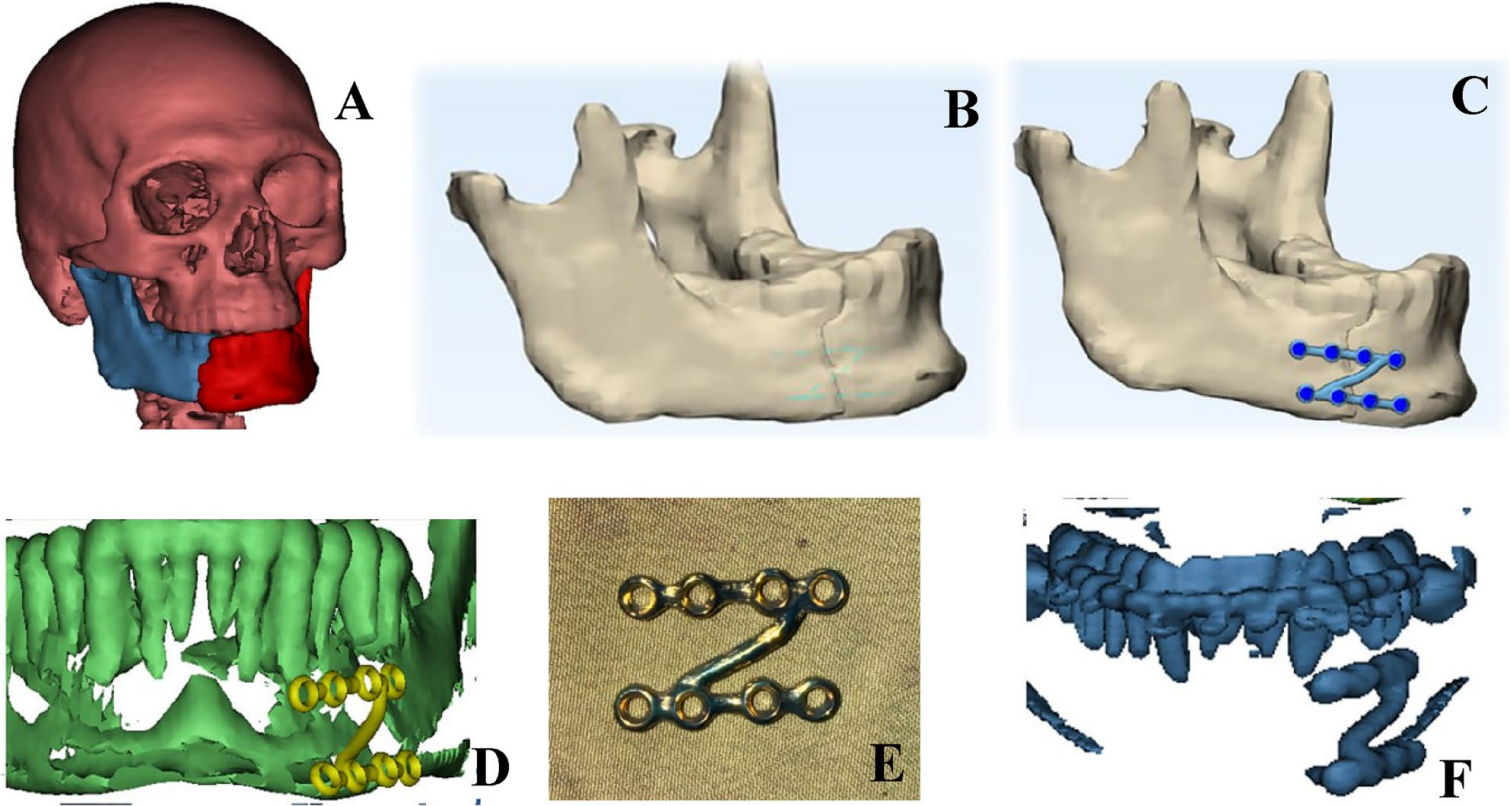
**A** 3D reconstruction of skull and fractured segments of mandible, **B** Reduction and union of fractured segment, **C** Planning of customized Z plate, **D** Root injury avoidance was checked preoperatively, **E** plate after milling and **F** postoperative 3D reconstructed mandible after reduction and fixation by customized Z- shaped miniplate revealed that upper arm of plate is away from teeth roots

Computed tomography (CT) DICOM file was imported into Mimics software (Mimics Research 21.0), followed by 3D reconstruction of skull and mandible then was imported at 3-matic Research 13.0 (x64). (Fig. 2A)

3-matic Research (13.0) was used for reduction of fractured segments based on healthy mirrored mandible (Fig. 2B), plate designing (Fig. 2C). Overlapping the plate design on 3D of skull at Mimics Research 21.0 was done to avoid root injury. (Fig. 2D and F). Final STL format of plate was sent for milling on a titanium block (Fig. 2E). Sterilization of milled plate.

##### Virtual planning for control group

The virtual reduction of mandibular fractures was done in the same way as study groups obtain a virtual reduced mandible.

#### Operative procedure

All patients treated under general anesthesia. Ivy loop or arch bar were applied. Fracture line exposed through intra-oral approach or the existing extra-oral wound, reduction guided by patient occlusion, maxillo-mandibular fixation (MMF).

**Fig. 3:**
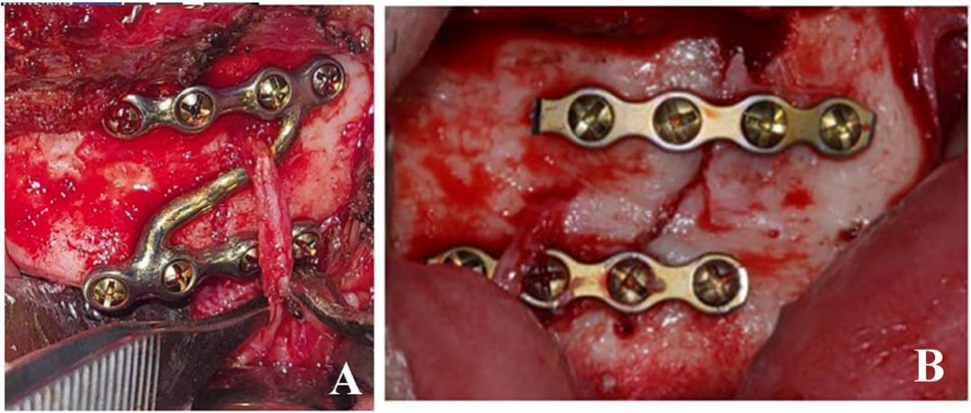
Fracture fixation by **A** customized Z- miniplate and **B** 2- miniplates

For group A: customized Z plate was used. (Fig. 3A)

For group B: conventional 2 miniplates were used. (Fig. 3B)

- In both groups, operation time was recorded from reduction to fixation only (Not involving preoperative time needed for virtual planning or MMF placement) to ensure standardized comparison.
- Standardized postoperative care, including ice pack application, prescribed medication andoral hygiene instruction for all patients.

#### Follow up phase

**Fig. 4:**
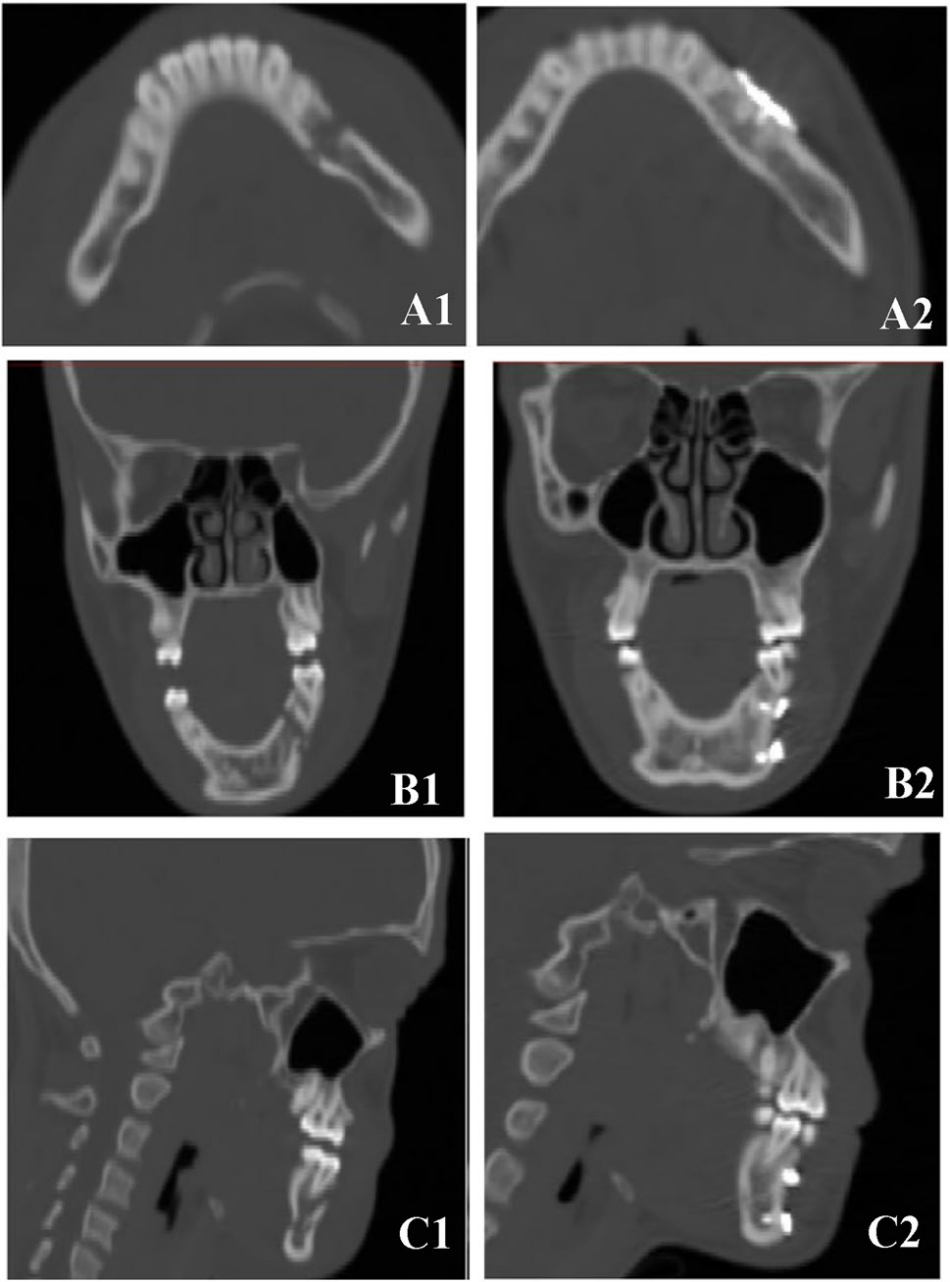
Preoperative vs. postoperative CT images (A1) Preoperative and (A2) Postoperative CT axial cuts. (B1) Preoperative and (B2) Postoperative coronal cuts. (C1) Preoperative and (C2) Postoperative CT sagittal cuts

The follow-up schedule for all patients in both groups was 24-hours, one, four, six, and twelve weeks postoperatively. CT for radiological evaluation of post operative symmetry and reduction accuracy was done within 2 days after surgery. (Fig. 4)

**Fig. 5:**
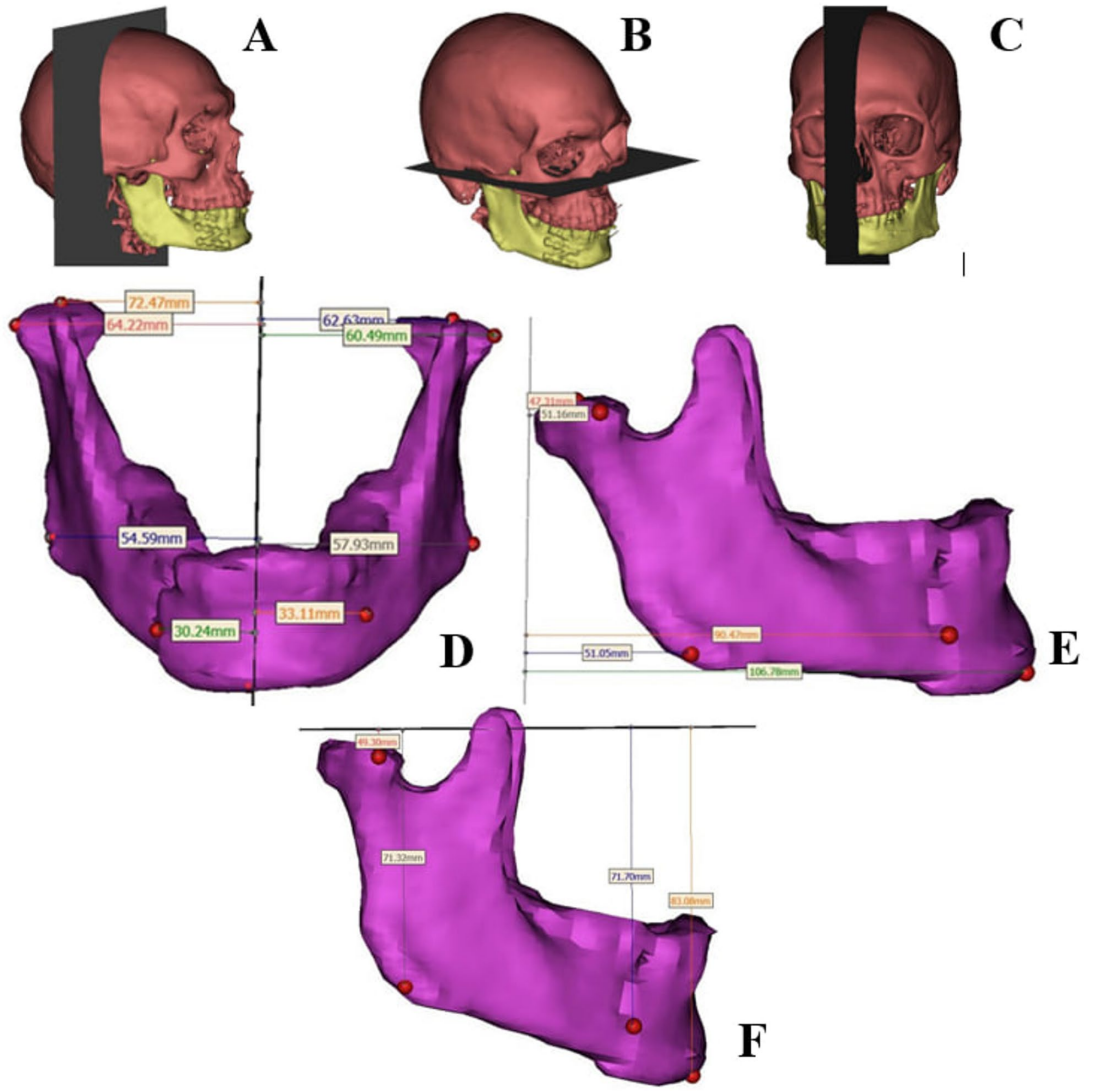
Demonstration of landmarks, planes and distance measurements used in this study. **A** Sagittal plane. **B** Axial plane. **C** Coronal plane. **D**–**F** Linear measurements between corresponding bilateral anatomical landmarks ( highest point of condyle (Co), lateral point of condyle (La), gonion (Go), mental foramen (MF) and Menton (Me))

Quantitative assessment of postoperative symmetry by technique developed by L. Zhao et al. [12]. Postoperative 3D models were reconstructed using Mimics software. Four mandibular points: (Co) The protrudest point on the top condyle, (La) The most protruding lateral point of condyle, (Go) The most inferior, posterior, and lateral point on the angle of the mandible, (MF) Mental foramen were identified bilaterally, and three planes: (MSP)mid sagittal plane, (TP)transverse plane, (CP)coronal plane were established. Vertical linear distances from each landmark to the reference planes were measured bilaterally, and the differences between corresponding measurements were calculated to assess postoperative symmetry. Lower values indicated greater symmetry. (Fig. 5)

Another method used to assess symmetry was the asymmetry index (AI) described by Huang et al. [13]. The AI for each landmark was calculated using the equation:

$\mathrm{AI}=\surd(\mathrm{LdS}-\mathrm{RdS})^2+(\mathrm{LdA}-\mathrm{RdA})^2+(\mathrm{LdC}-\mathrm{RdC})^2$, where L and R represent the left and right sides, respectively. The mean AI value was then calculated for each landmark in both groups.

Reduction accuracy was evaluated using three different digital methods. First, a quantitative method described by Zhao et al. [12] was applied by comparing the preoperative virtually reduced model with the postoperative model. Four landmarks (Co, La, Go, MF) bilaterally in addition to a midline landmark, menton (Me), were identified on both models. Three reference planes (MSP, TP and CP) were constructed as above (Fig. 5A–C). Vertical linear distances from the landmarks to the planes were measured (Fig. 5D–F), and the absolute differences between the virtual and postoperative models were calculated to assess reduction accuracy, where lower values indicated higher accuracy.

**Fig. 6:**
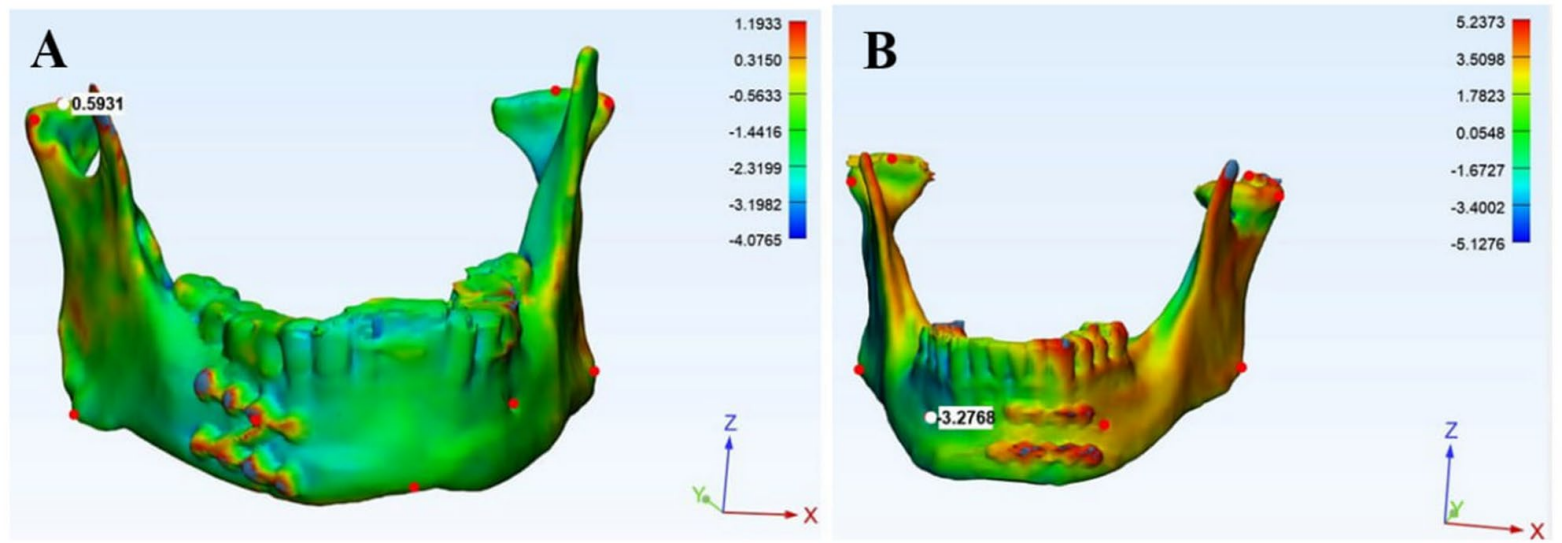
Three-dimensional surface deviation color-map analysis. **A** Error distribution across mandible fixed with customized Z miniplate; (**B**) Error distribution across mandible fixed with two-miniplate fixation; Both showed absolute local deviations were measured at bilateral landmarks (highest point of condyle (Co), lateral point of condyle (La), gonion (Go), mental foramen (MF) and Menton (Me))

The second method was three-dimensional surface deviation color-map analysis (part comparison analysis, PCA). Qualitative evaluation was performed by superimposing the postoperative model onto the virtually reduced model to generate a color-coded error map illustrating discrepancies between corresponding surface points (Fig. 6A, B). Quantitative analysis was then performed by measuring linear discrepancies (mm) at predefined bilateral landmarks (Co, La, Go, MF, and Me) using the Measure Analysis Locally tool.

Reduction accuracy was primarily interpreted using quantitative three-dimensional surface deviation color-map analysis derived from model superimposition (mean absolute deviation in millimeters). Postoperative symmetry was evaluated using the asymmetry index (AI), while other digital measurements were used as complementary assessments.

Adverse events were closely prospectively monitored at each visit, including infection, wound dehiscence, malocclusion, neurosensory disturbances, hardware-related complications, need for 2nd surgery, and radiation-related concerns.

### Statistical analysis

Normality of the continuous data was tested using the Kolmogrov-Smirnov and Shapiro-Wilk tests. Continuous data were described by the mean, standard deviation (SD), median, interquartile range (IQR), minimum, and maximum values. Categorical data were described by the frequency and percentage. Differences between Customized Z plates and the conventional 2 miniplates in surgical accuracy, facial symmetry and operation time were assessed by Mann-Whitney U test, while the comparison between the fractured and sound sides or between the preoperative and postoperative conditions in each group was assessed by Wilcoxon-signed rank test. Differences between Customized Z plates and the conventional 2 miniplates in associated fracture, fracture site and mode of trauma, were assessed by Chi-squared test with Fisher’s exact or Monte-Carlo corrections. Median difference, 95% confidence interval (CI), and Cohen’s d were calculated for the operative time. Median differences, 95% Hodges Lehman CIs, and Rank biserial correlation coefficients (r) for the primary reduction accuracy endpoint (surface deviation color map analysis), and the primary symmetry endpoint (Asymmetry Index) were calculated.

All analyses were conducted on an intention-to-treat analysis (ITT), as all patients completed the study according to the prespecified protocol without any deviations or loss to follow-up.

## Results

**Fig. 7:**
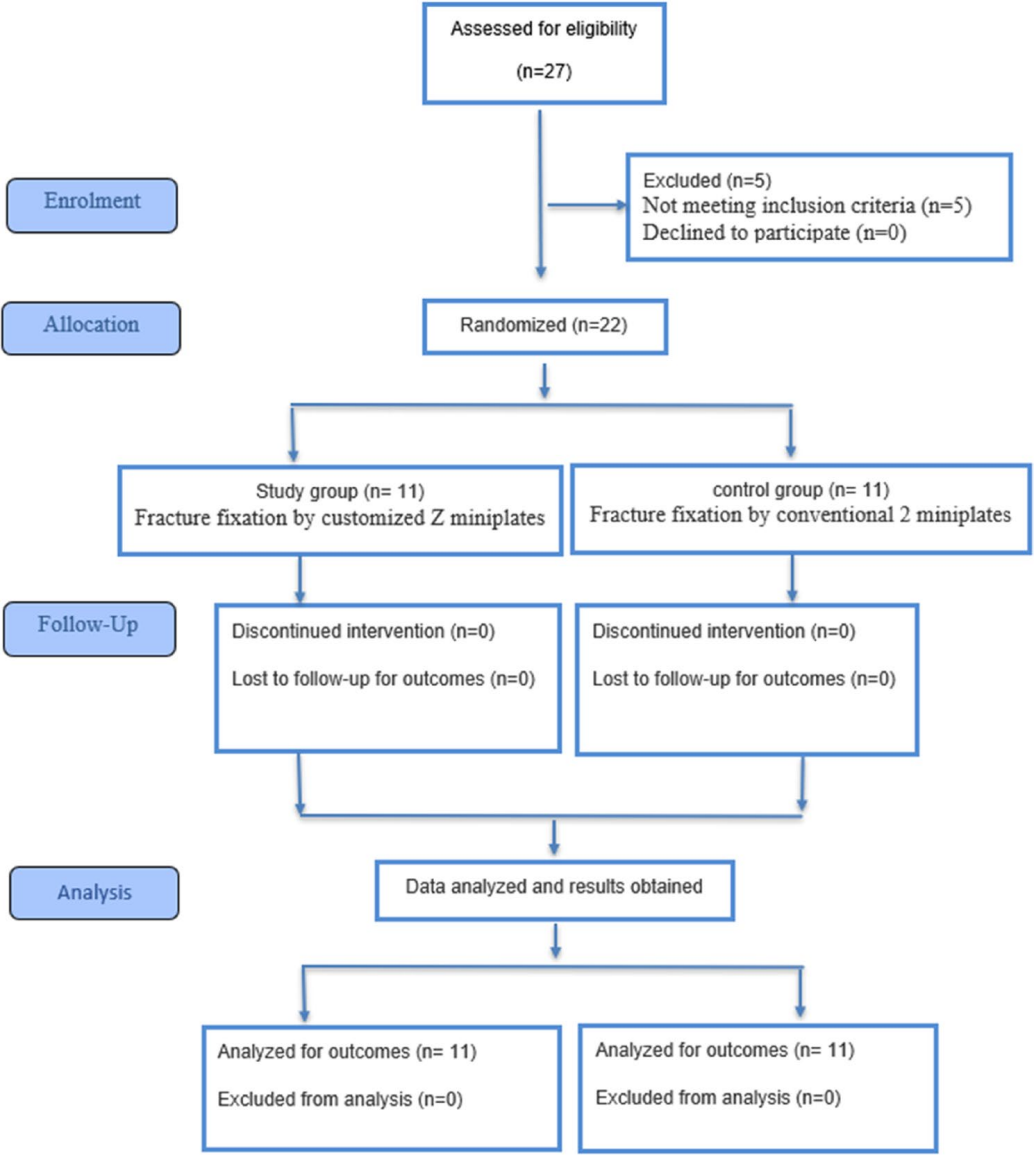
CONSORT flow diagram

All participants completed the 12-week follow-up. No participants were lost to follow-up, discontinued the intervention, or deviated from the study protocol. All participants were included in the final analysis (Fig 7).

A total of 22 patients (7 females and 15 males) with unilateral parasymphyseal or body mandibular fractures were recruited from the Emergency Department of Alexandria University Teaching Hospital between April and December 2024. Follow-up was done at the Oral and Maxillofacial Surgery Department, Faculty of Dentistry, Alexandria University, Egypt.

The age ranged from 20 to 42 years (mean 27.55 ± 6.68 in the study group and 29.09 ± 8.24 in the control group). Road traffic accidents were the most common cause of injury (*n* = 18), followed by falls from height (*n* = 3) and interpersonal violence (*n* = 1). Fractures involved either the mandibular body (*n* = 13) or the parasymphyseal region (*n* = 9). (Table 1)

**Table 1:** Description of the study sample

Variables		Customized Z plate *n* = 11	Conventional 2 miniplates *n* = 11	*p* value
Age	Mean (SD)	27.55 (6.68)	29.09 (8.24)	0.63^a^
	Min-Max	20.00–40.00	20.00–42.00	
Gender	Females N (%)	3 (27.27)	4 (36.36)	1.00^b^
	Males N (%)	8 (72.73)	7 (63.64)	
Associated fracture	Yes N (%)	4 (36.36)	5 (45.45)	1.00^b^
	No N (%)	7 (63.64)	6 (54.55)	
Fracture site	Body of mandible N (%)	7 (63.64)	6 (54.55)	1.00^b^
	Parasymphysis N (%)	4 (36.36)	5 (45.45)	
Mode of trauma	RTA N (%)	10 (90.91)	8 (72.73)	0.46^c^
	FFH N (%)	1 (9.09)	2 (18.18)	
	IPV N (%)	0 (0.00)	1 (9.09)	

*SD* Standard deviation, *Min* Minimum, *Max* Maximum, *RTA* Road traffic accident, *FFH* Falling from height, *IPV* Interpersonal violence

^a^Mann-Whitney U test

^b^Chi-square with Fisher’s-exact correction

^c^Chi-square with Monte-Carlo correction

The operation time for the study group ranged from 30.0 to 65.0 min with a mean of 44.64 ± 11.91 min, while for control groups ranged from 60 to 95 min with a mean of 78.64 ± 11.85 min. There was a statistically significant difference (*p* < 0.001) between the two groups where the operation time at the study group was reduced significantly than that of at the control group as shown in (Table 2).

**Table 2:** Difference between customized z plates and conventional 2 miniplates in operation time

Operation time (minutes)	Customized Z plate *n* = 11	Conventional 2 miniplates *n* = 11	Mean difference	Effect size (d)	95% CI	*p* value
Mean (SD)	44.64 (11.91)	78.64 (11.85)	-34.00	2.86	(-44.57, -23.43)	< 0.001*
Median (IQR)	45.00 (35.00, 55.00)	80.00 (65.00, 90.00)				
Min-Max	30.00–65.00	60.00–95.00				

Independent t-test

*SD* Standard deviation, *IQR* Interquartile range, *Min* Minimum, *Max* Maximum, *d* Cohen’s d, *CI* Confidence Interval

*Statistically significant at *p* < 0.05

Regarding the quantitative assessment of symmetry, the postoperative measurements showed that the differences were reduced and nearer to ZERO value for all the five landmarks on the three planes in study group compared to control group. These differences were significant statistically *p* < 0.05 indicating better facial symmetry for the customized Z Plate. In the study group postoperatively, mean value at sagittal plane was (Co is 2.97 mm, La is 2.18 mm, Go is 2.61 mm and MF is 2.52 mm), mean value at coronal plane (Co is 2.94 mm, La is 1.53 mm, Go is 2.45 mm and MF is 2.83 mm) and mean value at axial plane was (Go is 2.17 mm and MF is 1.78 mm). Similarly, for the control group postoperatively, mean value at sagittal plane was (Co is 7.39 mm, La is 5.96 mm, Go is 7.3 mm and MF is 6.29 mm), mean value at coronal plane (Co is 7.3 mm, La is 7.98 mm, Go is 6.85 mm and MF is6.73 mm) and mean value at axial plane was (Go is 8.26 mm and MF is 7.92 mm), as shown in (Table 3).

**Table 3:** Quantitative assessment of symmetry: between customized z plates and conventional 2 miniplates

Facial symmetry by the conventional test (mm)				Customized Z plate*n* = 11		Conventional 2 miniplates*n* = 11	*p* value ^a^
DS	Co	Postoperative	Mean (SD)	2.97 (1.86)		7.39 (2.96)	0.001*
			Min-Max	0.00–6.00		1.40–11.00	
	La	Postoperative	Mean (SD)	2.18 (2.29)		5.96 (2.85)	0.003*
			Min-Max	0.00–8.00		2.10–11.50	
	Go	Postoperative	Mean (SD)	2.61 (2.61)		7.30 (3.91)	0.001*
			Min-Max	0.00–9.00		2.50–15.00	
	MF	Postoperative	Mean (SD)	2.52 (2.37)		6.29 (4.26)	0.03*
			Min-Max	0.30–7.00		0.43–13.90	
DC	Co	Postoperative	Mean (SD)	2.94 (2.66)		7.30 (3.17)	0.004*
			Min-Max	0.00–7.30		2.90–13.40	
	La	Postoperative	Mean (SD)	1.53 (1.64)		7.98 (3.88)	< 0.001*
			Min-Max	0.00–6.00		2.02–16.00	
	Go	Postoperative	Mean (SD)	2.45 (2.48)		6.85 (4.45)	0.01*
			Min-Max	0.00–8.00		1.50–16.97	
	MF	Postoperative	Mean (SD)	2.83 (2.58)		6.73 (2.43)	0.002*
			Min-Max	0.30–9.00		2.90–10.50	
DA	Go	Postoperative	Mean (SD)	2.17 (1.88)		8.26 (4.14)	0.002*
			Min-Max	0.00–5.10		0.40–13.60	
	MF	Postoperative	Mean (SD)	1.78 (1.88)		7.92 (2.78)	< 0.001*
			Min-Max	0.00–5.50		4.02–12.50	

*SD* Standard deviation, *Min* Minimum, *Max* Maximum, *DC* Difference between linear points in coronal planes, *DS* Difference between linear points in sagittal planes, *DA* Difference between linear points in axial planes, CO Highest point of condyle, *La* Most lateral point of the condyle, *GO* most inferior lateral posterior part of angle of mandible, *MF* Mental foramen, *Ment* Menton

^a^Mann-Whitney U test

*Statistically significant at *p* < 0.05

Evaluating symmetry by AI, the mean AI was decreased for all the 4 landmarks in study group compared to control group indicating better symmetry. In the study group, mean value at Co, La, Go and Mf were (4.51, 4.31,8.3 and 7.99) respectively, while in control group, mean value at Co, La, Go and Mf were (9.37, 8.67, 11.03 and 10.7) respectively. The difference was significant statistically decreased for the point Co (*p* = 0.03) and La (*p* = 0.02), while this difference wasn’t statistically significant decreased for the points Go (*p* = 0.33) and MF (p value = 0.19), as shown in (Table 4).

**Table 4:** Symmetry assessment by Asymmetry Index (AI) between customized z plates and conventional 2 miniplates

Facial symmetry by AI (mm)			Customized Z plate *n* = 11	Conventional 2 miniplates *n* = 11	Median difference	95%CI	Effect size (*r*)	*p* value ^a^
Co	Preoperative	Mean (SD)	32.71 (8.48)	19.45 (10.51)	19.70	(8.64, 27.00)	0.72	< 0.001*
		Median (IQR)	37.00 (32.00, 43.83)	21.30 (10.00, 29.20)				
	Postoperative	Mean (SD)	4.51 (4.24)	9.37 (3.50)	-5.30	(-8.98, -0.20)	0.45	0.03*
		Median (IQR)	4.20 (0.00, 7.26)	9.00 (6.30, 12.30)				
Median difference			-33.00	-10.38	-			
95%CI			(-40.59, -25.70)	(-16.32, -3.62)				
Effect size (r)			0.89	0.88				
*p* value ^b^			0.003*	0.003*				
La	Preoperative	Mean (SD)	39.01 (9.47)	15.96 (8.59)	24.10	(14.60, 32.00)	0.78	< 0.001*
		Median (IQR)	36.57 (36.00, 47.50)	12.00 (9.90, 25.08)				
	Postoperative	Mean (SD)	4.31 (4.39)	8.67 (4.32)	-4.40	(-7.50, -1.40)	0.51	0.02*
		Median (IQR)	3.50 (1.60, 5.00)	7.92 (5.90, 9.50)				
Median difference			-35.23	-6.53				
95%CI			(-39.25, -29.13)	(-10.60, -3.58)				
Effect size (r)			0.89	0.88				
*p* value ^b^			0.003*	0.003*				
Go	Preoperative	Mean (SD)	49.17 (14.96)	20.01 (8.90)	27.22	(15.83, 40.30)	0.83	< 0.001*
		Median (IQR)	43.00 (36.70, 61.50)	22.00 (9.80, 27.17)				
	Postoperative	Mean (SD)	8.30 (5.15)	11.03 (5.52)	-2.75	(-7.34, 2.00)	0.22	0.33
		Median (IQR)	7.10 (4.45, 11.00)	11.10 (6.50, 13.50)				
Median difference			-37.71	-8.88	-			
95%CI			(-48.59, -31.10)	(-13.02, -5.05)				
Effect size (r)			0.89	0.88				
*p* value ^b^			0.003*	0.003*				
MF	Preoperative	Mean (SD)	40.25 (18.40)	22.75 (11.19)	15.92	(3.70, 28.70)	0.51	0.02*
		Median (IQR)	35.90 (27.00, 60.70)	20.00 (12.80, 33.35)				
	Postoperative	Mean (SD)	7.99 (5.80)	10.70 (3.09)	-2.40	(-7.80, 2.00)	0.29	0.19
		Median (IQR)	10.00 (2.90, 11.70)	11.56 (8.00, 12.40)				
Median difference			-29.70	-10.92	-			
95%CI			(-45.50, -20.40)	(-18.72, -5.90)				
Effect size (r)			0.89	0.88				
*p* value ^b^			0.003*	0.003*				

*SD* Standard deviation, *IQR* Interquartile range, CO Highest point of condyle, *La* Most lateral point of condyle, *GO* most inferior lateral posterior part of angle of mandible, *MF* Mental foramen, *Ment* Menton, *r* Rank biserial correlation coefficient

^a^Mann-Whitney U test

^b^Wilcoxon-signed rank

*Statistically significant at *p* < 0.05

Regarding quantitative assessment of accuracy of reduction, the values in the study group were statistically significant (*p* < 0.05) smaller than those in the control group indicating significantly better accuracy of reduction for the customized Z plate. In the study group at fractured side, mean value at sagittal plane was (Co is 1.46 mm, La is 1.6 mm, Go is 1.52 mm and MF is 0.9 mm), mean value at coronal plane (Co is 1.52 mm, La is 1.27 mm, Go is 1.5 mm, MF is 1.98 mm and Me 1.96 mm) and mean value at axial plane was (Go is 1.82 mm, MF is 1.54 mm and Me 1.45 mm). Similarly for the control group at the fractured side, mean value at sagittal plane was (Co is 11.83 mm, La is 14.04 mm, Go is 10.34 mm and MF is 11.01 mm), mean value at coronal plane (Co is 15.16 mm, La is 17.43 mm, Go is 16.7 mm, MF is 16.42 mm and Me 16.08 mm) and mean value at axial plane was (Go is 15.58 mm, MF is 14.95 mm and Me 13.9 mm), as shown in (Table 5).

**Table 5:** Surgical accuracy evaluated by the absolute value of difference between bilateral measurements at the virtual plan and postoperative models for both groups customized z plates and conventional 2 miniplates

CT linear measurements (mm)				Customized Z plate *n* = 11	Conventional 2 miniplates *n* = 11	*p* value ^a^
DS	Co	Fractured	Mean (SD)	1.46 (1.52)	11.83 (8.46)	0.01*
	La	Fractured	Mean (SD)	1.60 (0.99)	14.04 (9.35)	0.001*
	Go	Fractured	Mean (SD)	1.52 (1.32)	10.34 (5.34)	< 0.001*
	MF	Fractured	Mean (SD)	0.90 (0.69)	11.01 (8.42)	0.04*
DC	Co	Fractured	Mean (SD)	1.52 (1.42)	15.16 (3.93)	< 0.001*
	La	Fractured	Mean (SD)	1.27 (0.85)	17.43 (4.06)	< 0.001*
	Go	Fractured	Mean (SD)	1.50 (1.14)	16.70 (4.12)	< 0.001*
	MF	Fractured	Mean (SD)	1.98 (1.54)	16.42 (5.65)	< 0.001*
	Me		Mean (SD)	1.96 (1.26)	16.08 (7.62)	< 0.001*
DA	Go	Fractured	Mean (SD)	1.82 (1.48)	15.58 (5.45)	< 0.001*
	MF	Fractured	Mean (SD)	1.54 (1.06)	14.95 (4.08)	< 0.001*
	Me		Mean (SD)	1.45 (1.26)	13.09 (5.25)	< 0.001*

*SD* Standard deviation, *CT* Computed tomography, *DC* Difference between linear points in coronal planes, *DS* Difference between linear points in sagittal plane, *DA* Difference between linear points in axial plane, CO Highest point of condyle, *La* Most lateral point of the condyle, *GO* most inferior lateral posterior part of angle of mandible, *MF* Mental foramen, *Me* Menton

^a^ Mann-Whitney U test

*Statistically significant at *p* < 0.05

Finally, the surface deviation color map analysis quantitative assessment of accuracy of reduction revealed that the customized Z plate sstatistically significant (*p* < 0.05) outperformed the conventional 2 miniplates at all landmarks at fractured and sound side except for La point at fractured side where the difference is not statistically significant (0.30). In the study group at fractured side, mean value at Co is 0.16 mm, La is 0.18 mm, Go is 0.01 mm, MF is 0.01 mm and Me is 0.05 mm, while in the control group the mean discrepancies’ values were at Co is1.51 mm, MF is 1.29 mm, La is 1.33 mm, GO is 1.64 mm, MF is 1.29 mm and Me is 1.34 mm. (Table 6)

**Table 6:** Surface deviation color map analysis quantitative assessment of accuracy by superimposition of pre-operative virtually planned and postoperative models reconstructed from Computed Tomography for both customized z plates and conventional 2 miniplates groups

Surface deviation measurements (mm)			Customized Z plate *n* = 11	Conventional 2 miniplates *n* = 11	Median difference	95%CI	Effect size (*r*)	*p* value ^a^
Co	Fractured	Mean (SD)	0.16 (0.26)	1.51 (0.97)	-1.35	(-1.78, -1.05)	0.69	0.001*
		Median (IQR)	0.10 (0.01, 0.31)	1.45 (1.22, 1.93)				
	Sound	Mean (SD)	0.06 (0.17)	1.18 (0.94)	-1.45	(-1.76, -1.02)	0.54	0.01*
		Median (IQR)	0.07 (-0.10, 0.20)	1.56 (1.10, 1.86)				
Median difference			-0.08	-0.31	-			
95%CI			(-0.28, 0.09)	(-1.31, 0.45)				
Effect size (r)			0.38	0.19				
*p* value ^b^			0.21	0.53				
La	Fractured	Mean (SD)	0.18 (0.36)	1.33 (2.40)	-1.29	(-1.63, 0.61)	0.23	0.30
		Median (IQR)	0.10 (-0.03, 0.30)	1.49 (-0.60, 1.63)				
	Sound	Mean (SD)	0.07 (0.26)	1.38 (1.02)	-1.71	(-1.85, -1.33)	0.54	0.01*
		Median (IQR)	0.05 (-0.01, 0.20)	1.82 (1.69, 1.89)				
Median difference			-0.05	0.25	-			
95%CI			(-0.38, 0.09)	(-1.64, 1.31)				
Effect size (r)			0.15	0.51				
*p* value ^b^			0.61	0.09				
Go	Fractured	Mean (SD)	0.01 (0.15)	1.64 (1.06)	-1.66	(-2.06, -1.35)	0.69	0.001*
		Median (IQR)	-0.01 (-0.07, 0.10)	1.68 (1.30, 2.10)				
	Sound	Mean (SD)	0.21 (0.39)	1.23 (0.89)	-1.37	(-1.67, -0.72)	0.54	0.01*
		Median (IQR)	0.10 (-0.03, 0.50)	1.48 (1.23, 1.77)				
Median difference			0.12	-0.19	-			
95%CI			(-0.06, 0.55)	(-1.38, 0.41)				
Effect size (r)			0.36	0.28				
*p* value ^b^			0.23	0.35				
MF	Fractured	Mean (SD)	0.01 (0.25)	1.29 (1.01)	-1.64	(-1.90, -1.03)	0.55	0.01*
		Median (IQR)	0.10 (-0.02, 0.20)	1.66 (1.01, 1.95)				
	Sound	Mean (SD)	0.11 (0.38)	1.29 (0.95)	-1.56	(-1.82, -0.97)	0.54	0.01*
		Median (IQR)	0.04 (-0.05, 0.31)	1.74 (1.24, 1.87)				
Median difference			0.04	-0.05	-			
95%CI			(-0.14, 0.39)	(-0.36, 0.89)				
Effect size (r)			0.19	0.11				
*p* value ^b^			0.53	0.72				
Me		Mean (SD)	0.05 (0.26)	1.34 (0.95)	-1.61	(-1.86, -1.23)	0.55	0.01*
		Median (IQR)	0.02 (0.01, 0.10)	1.78 (1.30, 1.92)				

*SD* Standard deviation, *IQR* Interquartile range, CO Highest point of condyle, *La* Most lateral point of the condyle, *GO* most inferior lateral posterior part of angle of mandible, *MF* Mental foramen, *Me* Menton, *r* Rank biserial correlation coefficient

^a^Mann-Whitney U test

^b^Wilcoxon-signed rank, CI Hodges Lehaman confidence interval

*Statistically significant at *p* < 0.05

Surface deviation color map analysis qualitative assessment by superimposition of the virtual planning and postoperative models revealed a high degree of similarity between the reduced mandibular fractures and the virtual surgical plans in study group more than control group. The color mapping showed that most of areas of the mandible were green in the study group, which showed a highly successful operation with customized Z- plate. Figure 6 (A, B).

All linear measurements (Co, La, Go, MF, Me) demonstrated excellent intra-examiner reliability, with very high ICC values (≥ 0.99), narrow confidence intervals close to 1.0, and statistically significant P values (< 0.001). (Table 7)

**Table 7:** Intra examiner reliability of linear measurements

	**ICC**	**95% CI**	***P*** ** value**
Co	0.999	0.996, 1.00	< 0.001*
La	0.996	0.987, 0.999	< 0.001*
Go	0.997	0.991, 0.999	< 0.001*
Mf	0.995	0.983, 0.999	< 0.001*
Color map discrepancy	0.977	0.890, 0.995	< 0.001*

*ICC* Intraclass correlation coefficient

*Statistically significant difference at *p* value < 0.05

### Clinical outcomes and complications

Regarding neurosensory function, both subjective and objective assessments demonstrated progressive improvement and no persistent deficits at the final follow-up in both groups. Electrophysiologically in both groups, latency period remained stable without significant prolongation, conduction velocity was preserved and amplitude results showed conducive to nerve recovery without significant interference, indicating absence of adverse nerve impact related to the fixation technique.

Postoperative pain decreased significantly over time in both groups, reflecting normal healing progression rather than fixation-dependent differences.

No postoperative malocclusion was recorded in either group at any follow-up interval.

Regarding complications, minimal wound dehiscence was observed in one patient per group during the early postoperative period. These cases were attributed to inadequate adherence to postoperative instructions and were successfully managed by wound debridement, re-suturing and antibiotic coverage.

No hardware-related complications were detected and no secondary surgical correction was needed.

## Discussion

At the present study, there was a significant decrease in the operation time for study group with a mean of 44.64 ± 11.91 min, while for control with a mean of 78.64 ± 11.85 min. This is could be explained the customized plates provide better bone adaptation, no need for prepending and easier surgery so resulting in less operation time when compared with control group where the surgical reduction and fixation was dependent on the surgeon’s skills and experience [14]. This is similar with Troise et al. [15] where there was a clear decrease in the mean operational time from 78.64 ± 11.85 min for control group to 44.64 ± 11.91 min for study group.

Quantitative and qualitative assessments of symmetry and accuracy of reduction revealed that the use of customized Z-shaped miniplates was significantly effective in improving both.

Our results for symmetry demonstrated that the difference between all bilateral measurements for study group was significant, statistically less than control group (study group ranged between 1.53 and 2.97 mm; control group mean ranged between 5.96 and 8.26 mm) (Table 3), suggesting better symmetry at study group. The distances from Co and La to TP were extremely small, so these data were not included in comparison [12].

Evaluating symmetry by AI, the mean AI was decreased for all the 4 landmarks in study group compared to control group indicating better symmetry.

We concluded that better symmetry in study group results was due to the virtual planning and the use of customized plates. 3D virtual treatment planning offered a better understanding of the anatomy, nature and direction of displacement of the fractured bone segments.

Quantitative assessment of accuracy of reduction revealed that the values were statistically significant smaller at fractured side in the study group (study group mean ranged between (0.90 to 1.98 mm); control group mean ranged between 10.34 and 17.43 mm), indicating better accuracy of reduction for the customized Z plate. The distances from Co and La to TP and Me to MSP were very small, so these data were not included in comparison [12].

Results at the present study regarding symmetry and accuracy of reduction were not consistent with L. Zhao et al. [12] as there was no significant difference in post operative symmetry, surgical accuracy between the group that treated with prefabricated titanium plate and group treated with conventional ready-made plates, which was in similar with studies about the effects of prefabricated titanium plate for reconstruction of mandible [16].

This inconsistency could be explained by L. Zhao et al. [12] who reported that the management of comminuted mandibular fractures is difficult, so the group that treated with digitally planned reduction guides gave better results than other 2 groups where reduction guide plates were not used. While in the present study, we were dealing only with unilateral parasymphyseal/body mandibular fractures.

Accuracy of reduction assessed by surface deviation color map analysis quantitative and qualitative techniques revealed better results with customized Z plate. The difference was statistically significant at all points except for La point where p-value (0.30). This suggested that achieving ideal reduction of fractures at the posterior mandibular regions was more difficult. This may be due to the force applied by the plate, provided stability only on the fractured segments anteriorly while the condyles were influenced by their position in glenoid fossa. This is similar with Troise et al. [15] where the post-operative outcome in study group appeared to be closer to the ideal virtual planned reduction than the group with traditional surgery.

Zhou et al. [16] introduced another technique for surface deviation color map analysis quantitative assessment by determining ‘mean error’ referred to the average error value for all simulated mandibular model surface area sites and ‘standard deviation’ indicated the standard deviation for these error values for regional error values using 3-matic software, this helped to locate where the dominant error for the whole mandibular surface was distributed using an intuitive bar graph.

By comparing both techniques for surface deviation color map analysis quantitative assessment, technique that was used at our study was more accurate, as discrepancies between preoperative virtual reduced and postoperative model measured locally at 4 bilateral landmarks (Co, La, Go, MF) and menton point, while Z. Zhou et al. [16] measured mean error and standard deviation dominant error for the whole mandibular surface automatically by 3-matic software.

When the treatment results were assessed, there were some errors in the measurements despite the good results. This may be due to errors in any step at the digital process starting from obtaining the CT DICOM data (errors in image acquisition till errors during milling of the plates) [17, 18].

All the preoperative and postoperative virtual planning steps were done by one of the surgical team, this would be more relevant to surgical principles, save preoperative preparation time and operators may also feel more assured and less apprehensive [19].

Limitations of this study includes, First, small sample size and short the follow-up period, which mainly reflects early radiologic accuracy and short-term symmetry outcomes. Long-term stability and functional adaptation require extended follow-up.

Second, the study was conducted at a single center, which may limit the generalizability of the findings.

Third, only unilateral mandibular fractures were included. This design was selected to allow reliable comparison with the contralateral healthy side for symmetry and reduction accuracy assessment. Future studies should evaluate the performance of customized Z-shaped miniplates in bilateral mandibular fractures.

Finally, patient-reported outcomes were not included in this study. Future investigations incorporating patient-centered outcomes are recommended to provide a more comprehensive clinical evaluation.

Despite these limitations, the study was conducted on real-world trauma cases involving both genders and a relevant age range in a university hospital setting, which supports the clinical applicability of the findings to similar maxillofacial practice environments.

## Conclusion

Usage of the virtually customized Z- shaped miniplates showed significantly improved early postoperative reduction accuracy and symmetry, along with reduced operative time compared with conventional two-miniplate fixation. Within the limitations this study, customized Z-shaped miniplates appear to represent a promising and efficient fixation strategy for unilateral parasymphyseal/body mandibular fractures.

## Data Availability

The datasets used and/or analyzed during the current study are available from the corresponding author upon reasonable request.

## Abbreviations

CAD/CAM: Computer-aided design and computer-aided manufacturing
CI: Confidence interval
CT: Computed tomography
MMF: Maxillo-mandibular fixation
ORIF: Open reduction and internal fixation
PCA: Part Comparison Analysis
SD: Standard deviation
VAS: Visual Analogue Scale
3D: Three-dimensional
DC: Difference between linear points in coronal planes
DS: Difference between linear points in sagittal plane
DA: Difference between linear points in axial plane
CO: Highest point of condyle
La: Most lateral point of the condyle
GO: Most inferior lateral posterior part of angle of mandible
MF: Mental foramen
Me: Menton
ITT: Intention-To-Treat

## References

[1] Patrocínio LG, Patrocínio JA, Borba BH, Bonatti Bde S, Pinto LF, Vieira JV, Costa JM. Mandibular fracture: analysis of 293 patients treated in the Hospital of Clinics, Federal University of Uberlândia. Braz J Otorhinolaryngol. 2005;71:560–5. 16612514 10.1016/S1808-8694(15)31257-X PMC9441990

[2] Lee R, Robertson B, Manson P. Current epidemiology of facial injuries. Semin Plast Surg. 2002;16:283.

[3] Salam A. Pattern and management of mandibular fractures: a study conducted on 264 patients. Pakistan Oral Dent J. 2007;27:20–5.

[4] Breasted JH. The Edwin Smith Surgical Papyrus: published in facsimile and hieroglyphic transliteration with translation and commentary in two volumes. Chicago: University of Chicago Press; 1930.

[5] Motamedi MH. An assessment of maxillofacial fractures: a 5-year study of 237 patients. J Oral Maxillofac Surg. 2003;61:61–4. 12524610 10.1053/joms.2003.50049

[6] Buck G. Fracture of the lower jaw with replacement and interlocking of the fragments. Annalist NY. 1846;1:245.

[7] Kushner GM, Alpert B. Open reduction and internal fixation of acute mandibular fractures in adults. Facial Plast Surg. 1998;14:11–21. 10371890 10.1055/s-0028-1085298

[8] Michelet FX, Deymes J, Dessus B. Osteosynthesis with miniaturized screwed plates in maxillo-facial surgery. J Maxillofac Surg. 1973;1:79–84. 4520558 10.1016/s0301-0503(73)80017-7

[9] Farmand M, Dupoirieux L. [The value of 3-dimensional plates in maxillofacial surgery]. Rev Stomatol Chir Maxillofac. 1992;93:353–7. 1475603

[10] Kende PP, Wadewale M, Ranganath S, Desai H, Landge JS, Sarda A. An In Vitro Evaluation of a Novel Design Z Plate for Fixation of Mandibular Symphysis and Parasymphysis Fractures-A Finite Element Analysis. J Maxillofac Oral Surg. 2022;21:929–35. 36274868 10.1007/s12663-021-01576-3 PMC9474775

[11] Singh V, Puri P, Arya S, Malik S, Bhagol A. Conventional versus 3-dimensional miniplate in management of mandibular fracture: a prospective randomized study. Otolaryngol Head Neck Surg. 2012;147:450–5. 22647925 10.1177/0194599812449437

[12] Zhao L, Zhang X, Guo Z. Use of modified 3D digital surgical guides in the treatment of complex mandibular fractures. J Craniomaxillofac Surg. 2021;49:282–91. 33581958 10.1016/j.jcms.2021.01.016

[13] Huang CS, Liu XQ, Chen YR. Facial asymmetry index in normal young adults. Orthod Craniofac Res. 2013;16(2):97–104. 10.1111/ocr.12010. Epub 2012 Dec 4. PMID: 23324075. 10.1111/ocr.12010 23324075

[14] Liu XZ, Shu DL, Ran W, Guo B, Liao X. Digital surgical templates for managing high-energy zygomaticomaxillary complex injuries associated with orbital volume change: a quantitative assessment. J Oral Maxillofac Surg. 2013;71:1712–23. 23911146 10.1016/j.joms.2013.06.197

[15] Troise S, De Fazio GR, Committeri U, Spinelli R, Nocera M, Carraturo E, Salzano G, Arena A, Abbate V, Bonavolontà P, Romano A, Dell’Aversana Orabona G, Vaira LA, Piombino P. Mandibular reconstruction after post-traumatic complex fracture: Comparison analysis between traditional and virtually planned surgery. J Stomatol Oral Maxillofac Surg. 2025;126:102029. 39216729 10.1016/j.jormas.2024.102029

[16] Zhou Z, Zhao H, Zhang S, Zheng J, Yang C. Evaluation of accuracy and sensory outcomes of mandibular reconstruction using computer-assisted surgical simulation. J Craniomaxillofac Surg. 2019;47:6–14. 30471936 10.1016/j.jcms.2018.10.002

[17] Mueller CK, Zeiß F, Mtsariashvili M, Thorwarth M, Schultze-Mosgau S. Correlation between clinical findings and CT-measured displacement in patients with fractures of the zygomaticomaxillary complex. J Craniomaxillofac Surg. 2012;40:e93–8. 21733703 10.1016/j.jcms.2011.05.009

[18] Oka K, Murase T, Moritomo H, Goto A, Sugamoto K, Yoshikawa H. Accuracy analysis of three-dimensional bone surface models of the forearm constructed from multidetector computed tomography data. Int J Med Robot. 2009;5:452–7. 19722285 10.1002/rcs.277

[19] Li P, Tang W, Li J, Tian DW. Preliminary application of virtual simulation and reposition template for zygomatico-orbitomaxillary complex fracture. J Craniofac Surg. 2012;23:1436–9. 22948624 10.1097/SCS.0b013e318260edde

